# Ophthalmology and Surgery

**Published:** 1875-12

**Authors:** 


### II. Ophthalmology and Surgery.

1. *The Movements of the Iris.* A. Debouzy. (*La France Médicale*, Sept. 25, 1875.)

Debouzy admits the existence of radiating fibres, but denies their being antagonistic to the sphincter muscle. According to him all the muscular fibres, both radiating and circular, constitute a single muscular diaphragm supplied by the oculo-motor nerve. A simultaneous contraction of all the fibres produces a narrowing of the pupil, while the dilatation is due to the elasticity of the membrane. The sympathetic nerve exercises no influence upon the muscular fibres, but on the blood vessels of the iris, and it determines the size of the pupil by the depletion or fullness of these vessels, while the light does not influence this nerve. Experiments and clinical facts are stated in support of this theory.

2. *Puerperal Blindness.* W. J. Scott. (*Boston Med. and Surg. Reporter*, Oct. 9, 1875.)

During pregnancy or after delivery the sight is sometimes failing; and generally albumen is then found in the urine; but sometimes the kidney disease has passed away, while the visual disturbance continues. The ophthalmoscope shows a congestion of the retina, from which the patient may recover completely by judicious treatment. In the early stage the use of tincture of iron, with moderate doses of tinct. digitalis, is recommended. In the later period, strychnia, bromide of sodium or potassium, are indicated, to remove the long continued congestion.

3. *Treatment of Amblyopia Potatorum.* CHAS. S. BULL. (*Am. Jour. Med. Sci.*, July.)

The treatment of weak sight (amblyopia) occurring in persons addicted to strong drink, is by no means always successful, although the habit of drinking be given up. Many cases certainly recover if the patients observe total abstinence and receive some tonic medicine. Others, however, do not get well under these conditions, but improve when strychnia is administered hypodermically. But other cases, again, do not improve under the strychnia treatment. In these cases there are generally some other symptoms of chronic alcoholism, such as insomnia, obstinate dyspepsia and muscular tremor; and here Bull (like Galezowski, Quaglino, and others,) had some excellent results from the internal administration of the bromide of potassium. He usually commenced with ten grains three times daily, and increased each dose by five grains till the patient began to show some of the toxic effects, (sense of weight in the head, desire to sleep, weakness of memory). When this point is reached, it is better to omit the drug entirely for a few days, and then recommencing with a smaller dose than the highest one reached, gradually diminish the daily amount until the remedy can be discontinued entirely.

4. *Skin-Grafting.* HICKLE. (*Wiener Med. Wochenschr.*, 1875, No. 31; *Centralbl. f. Chir.*, 1875, No. 41.)

In the Rudolph Hospital (Vienna) the grafting was tried 1296 times on 62 patients during four years, and 842 attempts (65 per cent.) were successful. The experiment was tried on ulcers only that showed healthy granulations. The size of the grafts varied from that of a mustard seed to that of a bean; the smaller ones took best. For the first dressing a piece of tin foil was placed between the grafts and the plaster-strips; the limb was kept absolutely quiet. On changing the first dressing after twenty-four hours, the grafts showed a difference of color ; some were pale red and could not be washed off ; these had taken, and healed in ; others were of a deathly white color and could readily be washed away ; others again still showed the original color, and would, later, either heal in or drop off. The epidermis of the grafts was entirely shed off by the third day. The cicatrices formed by grafting had no greater power of resistance than those formed without it.

5. *Disturbance of Nutrition after Lesion of the Nerves of the Leg.* CHOUPPE (*Société de Biologie; Centralblatt d. Chir.*, 1875, No. 42.)

By the explosion of a bombshell a young man received a very ugly wound at the external and upper portion of the left leg ; a portion of the fibula became necrotic, and the healing of the wound took six months. For the next two years nothing abnormal was known excepting a slight diminution of the sensibility of the left leg. But after this time spontaneous ulcerations, without any tendency toward healing, broke out at the plantar side of the first and second toes and at the instep. And besides these ulcers there is now above the scar, a very hyperæsthetic spot of the size of a two-franc piece ; the slightest touch causes the most violent pain. Below the cicatrix there are several portions, which though insensitive to the touch, are the seat of very severe spontaneous pain. The first toe is completely anæsthetic, the second and third toes and a portion of the planta pedis are partially so. The growth of hair is abundant all over the leg, and its perspiration is very copious. The temperature of the left leg is always higher by many degrees than that of the right. The treatment has so far been unsuccessful.

6. *Treatment of Hydatid Disease of the Liver.* (*Lancet*, Sept. 18, 1875.)

The prevalent belief that simple tapping of a hydatid cyst in the liver is sufficient for its radical cure, owing to the death of the echinococcus following the escape of its fluid contents, is not entertained by M. Verneuil, the eminent surgeon attached to the Hôpital de la Pitié (*Revue de Thérapeutique Medico-Chirurgicale*, August 15). He believes that the remains of the cyst acting as a foreign body, if they do not suppurate themselves, yet set up suppuration in their vicinity. Recognizing also the danger of peritonitis from escape of the hydatid fluid into the abdominal cavity, he does not adopt the so-called method of Récamier—viz., external application of caustic—nor other means of promoting adhesion ; but he begins by puncturing the most prominent part of the cyst with a large trocar and canula. He then introduces through the canula a large gum-elastic catheter. Adhesions take place between the visceral and parietal peritoneum, and, there being constant means of escape, there is no retention of the fluid. Repeated injections of disinfectants, as solutions of carbolic acid or alcohol, are made into the cyst, the walls of which are thus allowed to slowly contract.

7. *Large Hydatid Cyst in the Abdomen.* MAAS. (*Memorabilien*, xx. 7.)

A merchant, 20 years of age, presented himself for operation, with the following symptoms: A moderate fullness and prominence of the right hypogastric region ; by palpation a fluctuating tumor could be traced from Poupart's ligament to within three centimetres below the navel ; percussion showed this tumor separated from both the liver and kidney by tympanitic spaces ; change of the patient's position did not alter the position of the tumor. Bladder sound, micturition normal. Simon's double puncture with two thin trocars was performed ; the trocars, four centimetres distant from each other, were thrust through the abdominal wall into the tumor to the right side of the arteria epigastrica ; the trocars remained in for three days, to cause an adhesion of the tumor to the abdominal wall ; then the bridge between the two punctures was cut through, and the whole cyst, of the size of a child's head, removed ; the membrane of the sac measured three to four millim. in thickness ; its fluid contained echinococci. The whole operation was performed under the antiseptic spray, the cavity washed out with carbolic acid, a drainage tube put in, and Lister's antiseptic dressing applied. No febrile reaction ensued, and the patient made a speedy recovery in 21 days.

8. *Severe Prolapsus Recti.* F. D. BEANE. (*Am. Jour. Med. Sci.*, October, 1875.)

"Astringent lotions, enemata, suppositories, trusses, are a delusion in cases of any moment; troublesome to the patients, unsatisfactory to the surgeon. Other operations, as snipping off a fold or two of skin at the verge of the anus, removal by ligature of pieces of the mucous membrane, Dupuytren's like removal by scissors, excision of a V-shaped piece of the sphincter ani, cauterization by nitric acid, are all greatly inferior to the operation by clamp and cautery, by reason of their *inefficiency*, hæmorrhage or subsequent bad results. The special advantages of this operation over nitric acid, are: 1. Absence of danger from abdominal inflammation; 2. Absence of hæmorrhage; 3. An immediate cure follows a well-performed operation; 4. Accuracy with which the amount of contraction of the mucous membrane may be determined. *No danger of subsequent stricture*; 5. Presence of firm vertical supporting cicatricial columns; 6. Lessened danger from absorption of septic material from sloughing surfaces." In support of these remarks the following case is presented: A married lady, æt. 42 years, had been afflicted with prolapsus recti for eight years. Walking a block or two, going up and down stairs, and, of course, the act of defecation, caused prolapse of the entire gut to the extent of three and a half inches. No stricture, uterus in normal position and healthy, stools hard and scybalous. Nitric acid was thoroughly applied in five broad vertical stripes the whole length of the prolapsed tissues. Ice, opium and perfect rest for five days, were required to relieve pain. After this, enemata of rhatany, liq. ferr. subsulph. were employed for several weeks, all without the slightest effect; defecation was attended by prolapse as before. March 29th, by means of Henry Smith's clamp-écraseur two anterior and two posterior vertical folds of mucous membrane (one and three-quarters inches in length) were successively taken up, half the tissue above the clamp was cut off and the rest was burned to the level of the instrument by the actual cautery; the parts anointed with carbolized oil were returned within the sphincter. Very slight febrile reaction and a dull ache in the rectum till the fifth day. Patient could walk the room on the twenty-first day, and has walked much ever since, but there has not been the slightest tendency to prolapse since the operation.

9. *Dislocation of the End of the Clavicle upon the Acromion of the Scapula.* H. F. Montgomery. (*Am. Jour. Med. Sci.*, October, 1875.)

The treatment of upward dislocations of the clavicle on the acromion has been entirely unsatisfactory, even in the hands of the ablest surgeons. Failure to retain the reduced bone in its socket has been the rule. The successful application of a new and simple method, and at the same time one readily tolerated by the patient, is illustrated by the following case: A blacksmith, aged 42 years, was thrown from a wagon, striking on his right shoulder. The external end of the right clavicle was dislocated from the acromion and resting upon it. The bone was readily reduced, but could not be retained in place by the various applications the doctor tried. He then concluded to try Prof. E. M. Moore's dressing for fracture of the clavicle. "Having again reduced the dislocation and keeping it in place by pressure upon the top of the external end of the clavicle, I carried the arm back and forced the elbow towards the left side, across the back, pressing the arm against the side of the chest. Directing a bystander to hold the arm in this position and to keep up pressure upon the end of the clavicle, I called for a common cotton sheet, which I folded cravatshape by placing the diagonal corners together, and then folding these corners over and placing them on the middle of the base line of the triangle, and again folding the sheet until the "cravat" is about four or five inches wide. The end of the bandage which is next to the body of the patient is carried by a half-spiral turn up along the front of the arm, in front of and over the injured shoulder, and then down across the back to the axilla of the sound side, carried forward under the sound arm, then upward in front of the sound shoulder, and over it to the back. The other end of the cravat is now carried around over the front of the elbow-joint and between the arm of the body to the back of the patient, then upward across the back to the top of the sound shoulder, there meeting the other end of the cravat. The ends were then drawn in opposite directions so as to make the bandage as tight as could be conveniently borne by the patient, and were fastened by sewing the lapped ends to each other. The hand of the injured side was supported by a sling fastened around the neck." On the twenty-first day of the treatment (which consisted in occasionally tightening the dressing) all dressings were removed and the treatment discontinued; there was not the least deformity, nor did the bone once move from its place after the first application of this dressing.

10. *Analogies of Dislocation of the Shoulder and Hip Joints.* KOCHER. (*Klinische Vortraege; Lond. Med. Record and Monthly Abst.*, Nov., 1875.)

Dr. Kocher bases the methods of reducing dislocations of the shoulder and hip on the anatomical structure of the joints. According to him, these joints possess several points of analogy, especially in their ligamentous apparatus. The Y-ligament of the hip-joint which proceeds from the anterior inferior spine to the linea intertrochanterica, and is connected with the zona orbicularis, has its analogue in the coraco-humeral ligament which, arising from the coracoid process, divides into two branches, one of which is inserted into the greater tuberosity, the other into the lesser tuberosity of the humerus. From both these branches fibres proceed to the capsule, and perform the same functions as the orbicular ligament. These anatomical analogies indicate a similar mode of reduction.

1. In dislocations upward and forward (sub-coracoid and ilio-pubic) the special movement for reduction is flexion, which in the case of the shoulder must be preceded by strong rotation outward, while in the case of the hip this has already been done. After flexion, rotation inward and extension follow.

2. Dislocations downward and forward (axillary and obturator) require rotation outward, which must be preceded by flexion and traction.

3. Dislocations downward and backward (infraspinous and sciatic) require rotation inward, flexion, traction, and finally rotation outward.

4. Dislocations upward and backward (sub-acromial and iliac) require flexion, or the utilization of that already existing, traction, and finally rotation outward.

The movements are essentially those of elevation and rotation. Elevation serves either for relaxation of the stretched portions of the capsule, or for stretching them so as to form a firm point for leverage.

11. *Varix.* RIGAUD. (*La France Médicale*, Sept.)

In operations for varicose veins of the spermatic cord and extremities, R. began by exposing the vein and cauterizing it with Vienna paste (Pruner, Strasburg, 1851). He noticed a considerable shrinkage of the veins when exposed to the atmosphere, and was thus led to try exposure without cauterization.

The vein is simply laid bare and isolated from contiguous tissues by a bit of black ribbon, a strip of plaster, or an India-rubber sound. About the seventh day the vein is commonly found obliterated. It gives way, and the parts heal rapidly. One hundred and forty cases of simple varix of extremities, and nineteen of varicocele, were thus successfully treated. In one case, the internal saphenous vein was implicated, with severe phlegmon and other serious complications. Success was hardly hoped for, but the termination was quite as happy as in the other instances.

